# Functional effects of drugs and toxins interacting with Na_V_1.4

**DOI:** 10.3389/fphar.2024.1378315

**Published:** 2024-04-25

**Authors:** Xinyi Zou, Zixuan Zhang, Hui Lu, Wei Zhao, Lanying Pan, Yuan Chen

**Affiliations:** ^1^ Zhejiang Provincial Key Laboratory of Resources Protection and Innovation of Traditional Chinese Medicine, College of Food and Health, Zhejiang Agriculture and Forestry University, Hangzhou, China; ^2^ Key Laboratory of Artificial Organs and Computational Medicine in Zhejiang Province, Shulan International Medical College, Zhejiang Shuren University, Hangzhou, China

**Keywords:** voltage-gated channel, skeletal muscle, Nav1.4, mexiletine, tetrodotoxin, drug design

## Abstract

Na_V_1.4 is a voltage-gated sodium channel subtype that is predominantly expressed in skeletal muscle cells. It is essential for producing action potentials and stimulating muscle contraction, and mutations in Na_V_1.4 can cause various muscle disorders. The discovery of the cryo-EM structure of Na_V_1.4 in complex with β1 has opened new possibilities for designing drugs and toxins that target Na_V_1.4. In this review, we summarize the current understanding of channelopathies, the binding sites and functions of chemicals including medicine and toxins that interact with Na_V_1.4. These substances could be considered novel candidate compounds or tools to develop more potent and selective drugs targeting Na_V_1.4. Therefore, studying Na_V_1.4 pharmacology is both theoretically and practically meaningful.

## 1 Introduction

Voltage-gated sodium channels (Na_V_) are crucial membrane proteins that control the electrical activity of cells involved in muscle movement and nerve signaling (Catterall, 2000; Ahern et al., 2016). These proteins are the main targets of various drugs, toxins, and disease-causing mutations affecting the cardiovascular and nervous systems (Sharan et al., 2015). The Na_V_ family in mammals consists of nine members, i.e., Na_V_1.1–Na_V_1.9. Each isoform is characterized by its electrophysiological and pharmacological properties, as well as its tissue expression pattern (Catterall et al., 2005). One of these members is Na_V_1.4, which is expressed in skeletal muscle cells and encoded by the *SCN4A* gene (MCClatchey et al., 1992). In excitable cells, Na_V_1.4 is responsible for initiating action potentials that trigger and regulate contraction in skeletal muscles. Therefore, any changes in this protein can have serious effects on muscle function. Na_V_ channels are composed of a single large α-subunit that forms the pore with one or two auxiliary β-subunits with an extracellular immunoglobulin (IG)-like domain and a transmembrane segment (OMalley and Isom, 2015). The β-subunits influence the trafficking and function of the α-subunit, which can form a Na^+^ channel by itself (Marban et al., 1998; Winters and Isom, 2016). The α-subunit has approximately 2,000 amino acids, including four similar transmembrane domains (DI–DIV). Each domain has six membrane-spanning α-helices (S1–S6) that are linked by intracellular loops. The S1–S4 segments make up the voltage-sensing domain (VSD) (Lee and Mackinnon, 2004; Decaen et al., 2008; Decaen et al., 2009), while the S5-S6 segments collectively form the pore module (PM) (MCCusker et al., 2012). Between S5 and S6, an α-helix re-entrant protrudes into the extracellular side of the membrane to create the narrow and asymmetric ion-selective filter (SF) (Ulbricht, 2005; Zhang et al., 2018). Four specific residues, Asp/Glu/Lys/Ala (DEKA), at the corresponding locus in the SF of each repeat, determine Na^+^ selectivity (Favre et al., 1996). The loop between DIII and DIV contains the fast inactivation gate with a hydrophobic IlePheMet (IFM) motif that plugs the intracellular mouth of the pore to stop the Na^+^ current following channel activation (Patton et al., 1992; West et al., 1992; Ulbricht, 2005). The C terminus (CTNa_V_; ∼200–300 amino acids in length) consists of a five-helix EF-hand-like motif (EFL, helices αI–αV) followed by a long α-helix (helix αVI) (Gardill et al., 2018) and extends into the cytoplasm of the cell where it interacts with several proteins, including the cellular calcium sensor calmodulin (CaM) (Qin et al., 2006).

**FIGURE 1:**
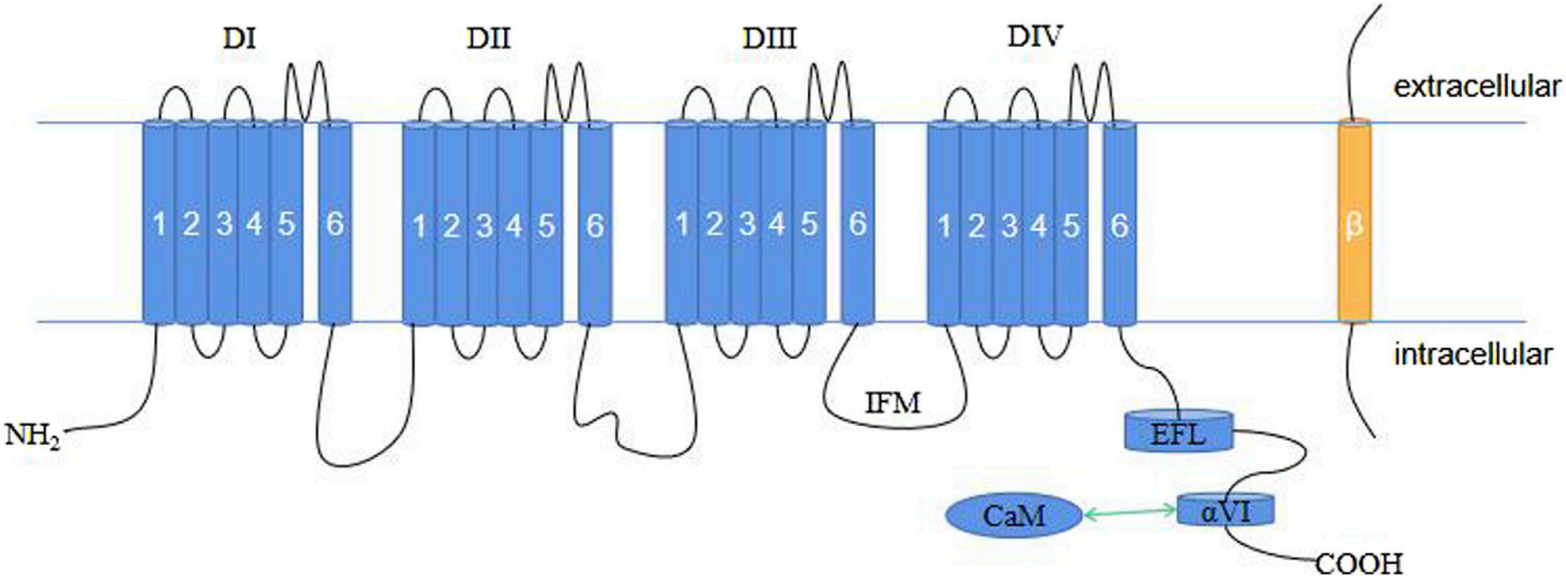
Schematic representation of the plane of the Na_V_1.4 structure. CaM, calmodulin; EFL, EF-hand-like motif; IFM, IlePheMet motif.

Na_V_ β-subunits are a group of proteins that are associated with Na_V_ channels. Na_V_ β-subunits comprise five subtypes in humans, namely β1–β4 and β1b, and are encoded by *SCN1B*–*SCN4B* (Eijkelkamp et al., 2012). These subunits have a single transmembrane structural domain (β1b deletion) and an IG-like extracellular domain with a molecular mass of approximately 30–40 kDa (Sanchez-Solano et al., 2017). The β1 and β3 subunits interact noncovalently with the α-subunit of the Na_V_ channel, whereas the β2 and β4 subunits are covalently bound to the α-subunit via disulfide bonds. Na_V_ channel β-subunits influence Na_V_ channel function through multiple mechanisms, including modulating their expression on the plasma membrane, affecting their gating properties, mediating cell–cell adhesion, and interacting with other proteins (OMalley and Isom, 2015; Calhoun and Isom, 2014; Angsutararux et al., 2021) (Figure 1).

Na_V_1.4 channels have a complex gating mechanism that involves resting and opening processes. Na_V_ channels can switch between at least three different states, and possibly more, including closed (resting), open, and inactivated (Catterall et al., 2020). According to the state-dependent blocking theory, sodium channel blockers have varying affinities for distinct conformations and functional states of the channels. Therefore, understanding the pharmacological action and mechanism of drugs and toxins that target the Na_V_1.4 channel and obtaining a comprehensive knowledge of the gating mechanism of Na_V_ channels are important (Deuis et al., 2017). When the Na_V_ channel is in the resting or closed state, all four VSDs may be in the “down” state. When the membrane is depolarized, the channels are activated, and they quickly open, allowing the pore domain to connect through S4-S5. This change in the channel conformation allows sodium ions to pass through the pore; however, channel opening is followed by rapid inactivation, which results in a non-conducting inactivated channel state, which then eventually recovers from inactivation back to the resting state (Guy and Seetharamulu, 1986). The rapid inactivation is mediated by the IFM residue, which are the residues of the S4-S5 linker binding the pore domain, thereby closing the channel (Chanda and Bezanilla, 2002; Payandeh et al., 2011).

Na_V_1.4-associated channelopathies are dominant diseases that affect skeletal muscle excitability and are classified into two opposite groups: non-dystrophic myotonia (NDM) and periodic paralysis (PP), defined by their prevalent clinical symptoms (Nicole and Fontaine, 2015). NDMs are conditions that cause muscle stiffness upon voluntary movement due to delayed skeletal muscle relaxation. This group includes sodium channel myotonia (SCM) and paramyotonia congenita (PMC). PP is characterized by episodic muscle weakness often related to potassium levels. It includes hyperkalemic (HyperPP) and hypokalemic periodic paralysis (HypoPP) (Cannon, 2018). Table 1 presents the main categories of Na_V_1.4 skeletal muscle channelopathies, describes the clinical phenotypes, triggers, Na_V_1.4 mutations and newly identified pathogenic mutations, as well as the clinically preferred therapeutic agents associated with muscle disorders.

**TABLE 1:** Clinical phenotypes associated with Na_V_1.4 mutations (Loussouarn et al., 2015; Maggi et al., 2021).

	Diseases	Function	Triggers	Specific feature	New mutations	Treatment
NDM	SCM	GOF	Cold	Skeletal muscle stiffness after voluntary contraction, without muscle atrophy	L703P Ke et al. (2022)	Mex
					V445M Huang et al. (2020)	
	PMC	GOF	Cold	Paradoxical myotonia	V781I Lee et al. (2022)	Mex
			Exertion		A1737T Lee et al. (2022)	
PP	HyperPP	GOF	Rest after exercise	Episodes of flaccid paralysis, leading to muscle weakness, generally associated with ictal hyperkalemia (>4.5 mEq/L)	V792G Segawa et al. (2023)	ACZ
			Fasting			
			Cold exposure		I692M-S906T Fan et al. (2017)	
	HypoPP2	GOF	Glucide-rich meals Rest after exercise Prolonged rest	Episodes of focal (limb) or more frequently generalized flaccid paralysis, with concomitant hypokalemia (<3.5 mEq/L)	R672/G/H/S Sternberg et al. (2001)	ACZ
CMS		LOF	Gene mutations	Predominant axial and pelvic muscle weakness, delayed motor milestones, improvement in strength over time	R1454W Berghold et al. (2022)	ACZ

SCM, sodium channel myotonia; PMC, paramyotonia congenita; HyperPP, hyperkalemic periodic paralysis; HypoPP2, hypokalemic periodic paralysis type 2; CMS, congenital myasthenic syndrome; GOF, gain-of-function; LOF, loss-of-function; Mex, mexiletine; ACZ, acetazolamide.

In this review, we summarize the ion channel diseases caused by Na_V_1.4 gene mutation, as well as clinical treatment options, drugs and toxins sensitive to Na_V_1.4 channels. We focus on the binding sites of this channel with these drugs and toxins, and analyze how they regulate the activity of Na_V_1.4. The development and research progress of new drugs for the treatment of Na_V_1.4 channel diseases are further discussed. The purpose of this review is to have a more comprehensive and macroscopic understanding of the pharmacological properties of human Na_V_ channels and Na_V_1.4, and to provide new treatment ideas and directions for these channel diseases.

## 2 Sodium channel blockers for the treatment of NDM

A possible method of managing NDM is to administer sodium channel blockers that can lower the excessive firing of action potentials in overactive muscle fibers. These sodium channel blockers used in clinical settings include anti-arrhythmics (De Bellis et al., 2017), local anesthetics (LAs), and antiepileptics (De Bellis et al., 2023); however, these are not selective for Na_V_1.4 and can affect all sodium channels. Therefore, improving the understanding of molecular binding mechanisms and structural designs to identify more effective compounds with fewer side effects that can target Na_V_1.4 is crucial (Peschel et al., 2020).

### 2.1 Anti-arrhythmics, the most commonly used treatment for NDM in clinical practice

**FIGURE 2:**
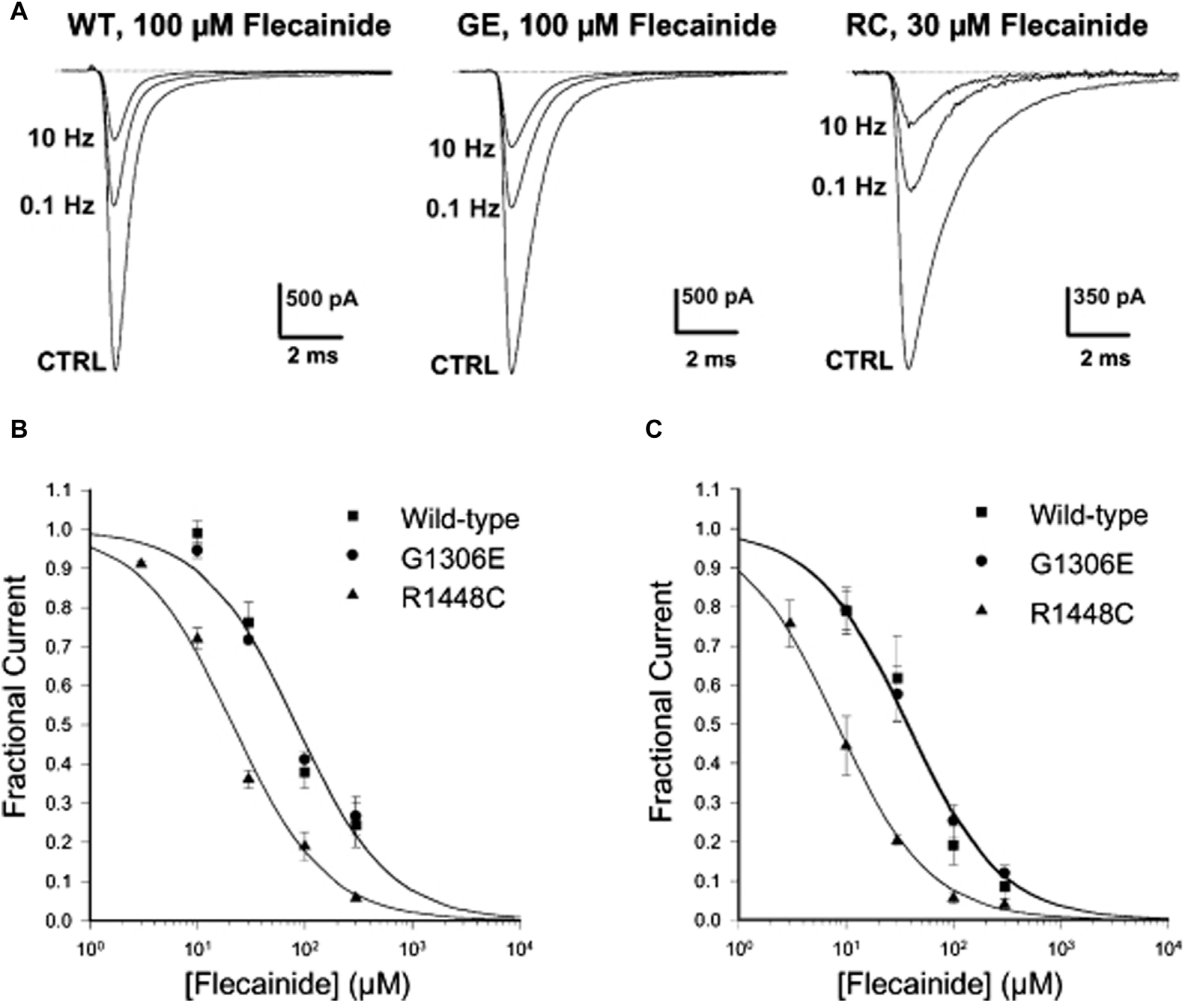
Frequency-dependent flecainide blockage of wild-type (WT) and mutant hNa_V_1.4 channels at a holding potential of −120 mV. **(A)** Three minutes after treatment with flecainide, the Na current blockage of WT and mutants was evaluated under different stimulation frequencies. **(B,C)** Concentration-response fitting curve of flecainide on WT and mutants blockage at stimulation frequencies of **(B)** 0.1 Hz and **(C)** 10 Hz. (Copyright permission was obtained for the use of these images.) CTRL, Control; GE, G1306E; RC, R1448C (Desaphy et al., 2004). (Order Number:5734610752304).

Anti-arrhythmic drugs belong to sodium ion channel blockers and are widely used in clinical practice. These blockers are also used to treat NDM. Anti-arrhythmic drugs block Na currents (*I*_*Na*_) in a use-dependent manner. Use-dependent refers the drugs are more effective when the muscles are in use or related ion channels are in activation or inactivation state. As result, they can reduce the action potentials triggered by muscle stiffness and the frequency of muscle membrane depolarization, thus achieving therapeutic effects. Therefore, the higher frequency of muscle membrane depolarization (e.g., muscle stiffness) and the faster heart rate (e.g., ventricular fibrillation), the greater blocking effect appear, with anti-arrhythmic drugs exerting effects specifically on the muscle-tonic discharge of the action potential (Desaphy et al., 2021). Mexiletine (Mex), a class IB anti-arrhythmic medicine, is considered as the first-line drug for treating NDM among anti-arrhythmic drugs (De Luca et al., 1997). In 2012, a randomized, double-blind, placebo-controlled 2-period crossover study on Mex in the treatment of 59 NDM patients was published (Statland et al., 2012), which showed that the use of Mex improved varying degrees of stiffness in patients after 4 weeks of treatment. This result also confirms the above viewpoint. It binds mainly to DIII S6 and DIV S6 in the pore domain structure of the sodium channel, which extends its refractory period by delaying the recovery from the inactive state (De Bellis et al., 2017). This also explains that Mex, like the muscle relaxant methylamine, has a dose-dependent effect on inhibiting resting muscle spindle discharge at low concentrations (Zhang et al., 2021a; Watkins et al., 2022). Interestingly, regardless of the origin of the gene mutation that causes NDM, it has a certain efficacy; however, mutations themselves can alter the sensitivity of this channel to Mex (De Bellis et al., 2023). For example, in 2012, two patients with NDM and severe neonatal paroxysmal laryngospasm (SNEL) with the *SCN4A* G1306E mutation (Figure 2) responded well to Mex administration (Caietta et al., 2013). In a study back in 2001, it was indicated that the G1306E mutation may reduce the blocking effect of Mex (Desaphy et al., 2001). Nevertheless, Mex remains the primary drug for the treatment of NDM on the long-term safety and Mex also confirms its important role in the treatment of NDM diseases (Suetterlin et al., 2015).

To design better Na_V_1.4 selective blockers, there is a need to understand the structure of compounds that block Na_V_1.4 more specifically. One common approach is to use Mex-like analogs that modify the spatial site resistance of Mex to the binding site as templates for other sodium channel blockers (De Bellis et al., 2013). For example, compared with Mex, sulfurized and chlorinated compounds with a lipophilic aromatic phenyl group substituted for the methyl group on the asymmetric carbon atom of Mex are 10- and 20-fold more effective in producing the tonic blocks, respectively. Due to the increase in lipophilicity, their use-dependence is reduced (De Luca et al., 2003). However, Mex and its analogues often cause side effects such as dyspepsia, which limits their clinical dosage and sometimes makes them unsuitable for some patients with NDM (Modoni et al., 2020), who may need other sodium channel blockers as alternative treatment.

Tocainide is a class IB anti-arrhythmic drug. It is also one of the few drugs that can reduce the symptoms of tonic syndrome, a condition that causes muscle stiffness and spasms. Some studies have reported that tocainide can effectively treat patients with PMC, a rare form of tonic syndrome, at low doses (Streib, 1987). However, tocainide poses a high risk of severe side effects such as agranulocytosis and anemia (Soff and Kadin, 1987); thus, there is a need to develop new variations of tocainide that are safer and more specific for antimyotonic purposes. One way to achieve this goal is to modify the proline part of tocainide, in which the asymmetric carbon atoms are constrained to rigid α-proline cycle. The tocainide derivative, To5, is 5 and 21 times stronger than tocainide in producing tetanic and 10 Hz-use-dependent blockade of skeletal muscle sodium current, respectively, and has better therapeutic potential. (Talon et al., 2001). N-benzylated β-proline derivatives have been proved to be the most effective use-dependent blocker of heterologous expression of hNa_V_1.4; therefore, determining the sites and characteristics of its specific binding with Na_V_1.4 is crucial for exploring potential candidate drugs in the existing Pharmacopoeia (De Luca et al., 2012).

Flecainide, often used for its blocking effects on the cardiac Na^+^ channel, belongs to the IC type of anti-arrhythmic drugs similar to Mex (Nitta et al., 1992). A case of a girl with the *SCN4A* G1306E mutation and SNEL who did not respond to Mex but improved with flecainide was reported in 2016 (Portaro et al., 2016). Desaphy et al. (2004) showed that flecainide could block sodium ion currents in SCN4A wild-type (WT), G1306E, and R1448C (PMC) mutations in an interdependent manner, but it is more effective against R1448C (Figure 2). This phenomenon also confirmed that even though flecainide and Mex exhibit the same blocking mechanism of the skeletal muscle sodium channel, drug selection could be determined by the gating defect caused by individual mutations, especially the specific voltage dependence of sodium channel availability.

In 2007, propafenone, a different type of IC anti-arrhythmic medication, was shown to be effective in treating patients with PMC and significantly reduced the clinical symptoms of cold-induced muscle stiffness (Alfonsi et al., 2007). Subsequently, Farinato et al. (Farinato et al., 2019) pharmacologically characterized tonic Na_V_1.4 mutations using different drugs and found that most mutations had decreased sensitivity to Mex and, unexpectedly, did not have an altered response to flecainide and propafenone. However, some clinical results raised the possibility of a link between skeletal muscle and cardiac sodium channelopathies. Therefore, anti-arrhythmic drugs such as flecainide and propafenone should be used with caution in patients with myotonia (De Bellis et al., 2017).

### 2.2 LAs have great potential for the treatment of NDM

The analysis of the structure from crypto-electron microscope has revealed that, the same as other subfamily of Na_V_ channels, Na_V_1.4 channel has VSD and pore domain, which is structured by its four S6 segments from its four different domains (Pan et al., 2018). LAs with a hydrophobic ring and an alkaline amine can penetrate the cell membrane and block the channel from inside of cells by binding the pore region to achieve its tonic inhibition on Na_V_1.4 channel. In 2005, Lipkind and fozzard (Lipkind and Fozzard, 2005) conducted molecular modeling of Na_V_1.4 and LAs, revealing LAs could bind with Leu-1280 (DIII S6) and Phe-1579 of DIV S6 with its alkaline amine. In addition, tits aromatic ring could interact with Tyr-1586 of DIV S6 and Asn-434 of DI S6. Further research has found that LAs have different blocking effects on different Na_V_ channels. Scholz group found that TTX sensitive (TTX-s) Na_V_ channels, which can be blocked by TTX and their IC_50_s of TTX is in single digit nano-molar range, were more susceptible to LAs than TTX resistant (TTX-r) Na_V_ channels including Na_V_1.8 and Na_V_1.9. For example, the half maximum inhibitory concentration (IC_50_) of lidocaine for tetanic block of TTX-r Na^+^ current is 5 times stronger than that of TTX-s (Scholz et al., 1998). In 2016, Gingrich and Wagner group (Gingrich and Wagner, 2016) reported that lidocaine regulated the Na^+^ current of rNa_V_1.4 (rat Na_V_1.4) channel by blocking the channel at open state, which is the second high affinity block comparing with the highest affinity block at inactivation state. Although there are few examples and literatures on the clinical use of LA drugs (LAs) in the treatment of NDM, there is no doubt that they have effects on NDM as LAs are sodium channel. We speculate the side effects of LAs preventing its clinic application of NDM. Through in-depth study of the interaction mechanism between local anesthetics and Na_V_1.4 channel, we can still be benefit by better understanding its mechanism of action, which provides more theoretical support for clinical practice and new drug development.

### 2.3 Antiepileptics have therapeutic effects on patients with Mex intolerance

**FIGURE 3:**
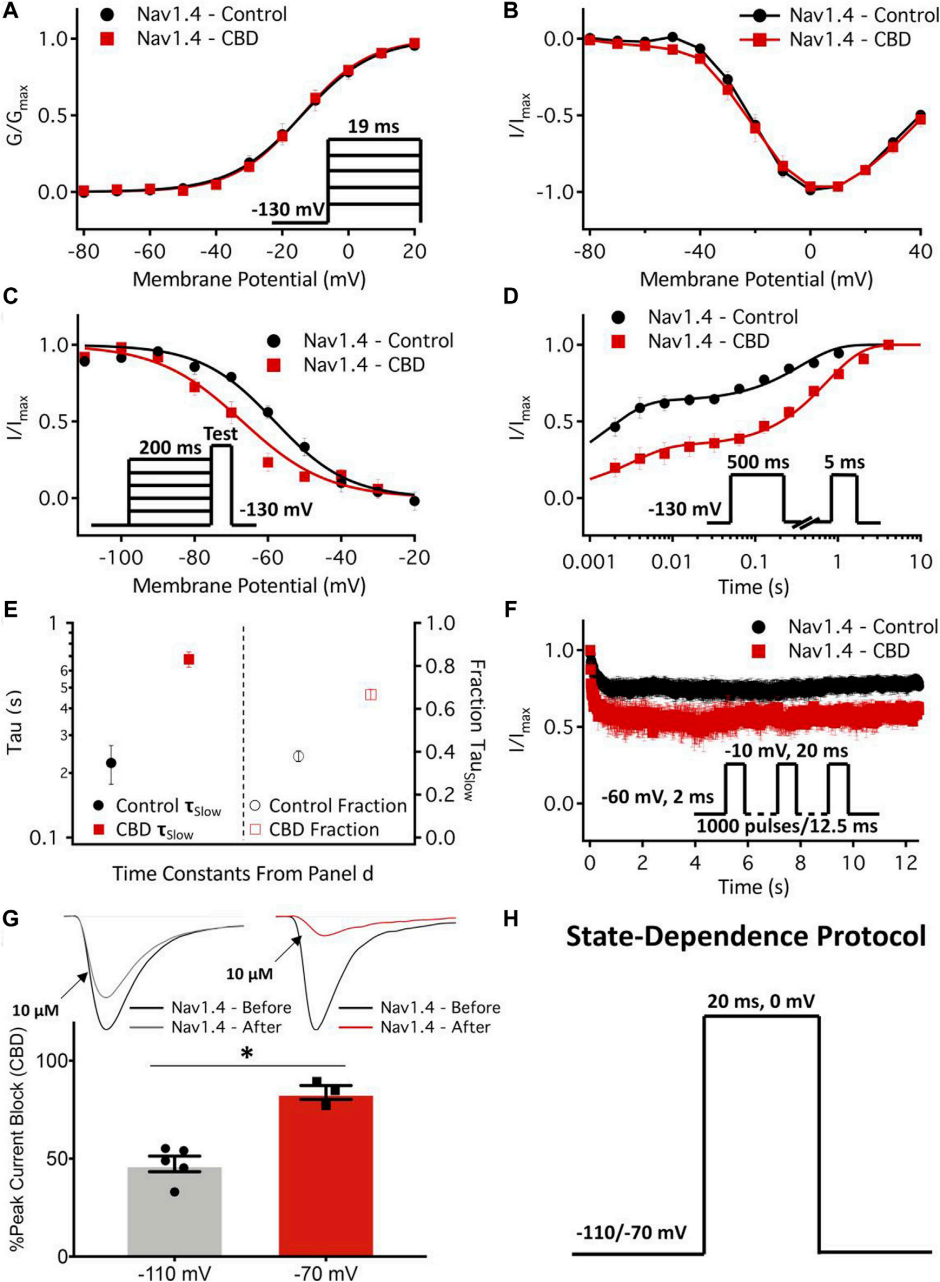
Effects of cannabidiol (CBD) on Na_V_1.4 gating. **(A,B)** Voltage dependence of activation shown as normalized conductance plotted against membrane potential in 1 µM CBD, and normalized activating currents as a function of potential. **(C)** Voltage dependence of 200 ms F-I curve plotted against the membrane potential in 1 µM CBD. **(D)** Recovery from fast inactivation in 1 µM CBD at 500 ms. **(E)** The slow components of recovery from inactivation in the control and CBD (1 µM) at 500 ms are shown on the left y-axis (logarithmic scale), and the fraction of the slow-to-fast component of recovery from inactivation is shown on the right y-axis. **(F)** Use-dependent inactivation in control and 2 µM CBD. The normalized current decay is plotted as a function of time fitted with an exponential curve. **(G)** State-dependent block of the peak Na_V_1.4 current at 10 µM. **(H)** Pulse protocol used for state dependence experiments. Recordings were performed at 1 Hz. (Copyright permission was obtained for the use of these images.) (Ghovanloo et al., 2021) (Order Number: 1453352).

Antiepileptic drugs are usually neutral in charge, unlike LA drugs, which are primarily cationic. However, both antiepileptic and LA drugs can interact with critical residues in the DIV S6 of Na_V_1.4 (Ragsdale et al., 1996) and block the channel with similar affinity regardless of state, i.e., open or inactive. This hypothesis was confirmed by Buyan et al. (2021), who used molecular dynamics (MD) simulations to compare the binding modes of the two drug classes. They also discovered that the Y1593 residue in Na_V_1.4 was essential for drug binding (Lipkind and Fozzard, 2010). The blockade mechanism involves the aromatic ring of antiepileptics almost perpendicularly inserting into the pharmacophore, occupying the pore cavity. This may cause interactions with other S6 fragments and physically obstruct the inner pore, preventing Na^+^ from entering (Lipkind and Fozzard, 2010). Phenytoin, carbamazepine, and lamotrigine are relatively common antiepileptic drugs. Lamotrigine has a particularly good therapeutic effect for patients who do not respond or are intolerant to Mex, as shown by clinical trials (Andersen et al., 2017; Vereb et al., 2021). Other antiepileptics, such as lacosamide and rufinamide, can also reduce myotonia in isolated human and rat skeletal muscles, but they have different inhibitory concentrations (Skov et al., 2017). In 2021, a cannabinoid from the cannabis plant, cannabidiol (CBD), was reported to relieve myotonia caused by sodium channelopathy, especially when the channel is in its slow inactivation state, owing to its high binding affinity for Na_V_1.4 channels (Huang et al., 2021). Ghovanloo et al. (2021) also studied the localization of CBD in the membrane using MD simulation and nuclear magnetic resonance and found that CBD could reduce the excitability of the Na_V_1.4 P1158S mutant (associated with NDM and PP). This suggests that CBD may have therapeutic potential for ion channel diseases with Na_V_1.4 hyperexcitability (Ghovanloo et al., 2021) (Figure 3).

## 3 Pharmacological treatment of primary PP caused by the *SCN4A* mutation

Primary PP is a rare autosomal dominant genetic disorder that affects the sodium channel Na_V_1.4 gene in skeletal muscle, leading to Hyper- or HypoPP. These conditions are characterized by episodes of muscle weakness or paralysis triggered by changes in potassium levels or other factors. The muscle membrane becomes depolarized, and the sodium channel becomes inactive, reducing the ability of muscles to contract (Finsterer, 2008). The current treatment options are mainly preventive and symptomatic, involving dietary and lifestyle changes, potassium supplementation or restriction, and drugs such as carbonic anhydrase inhibitors (CAIs), including acetazolamide (Sansone et al., 2008) and diclofenamide (Sansone et al., 2016), and diuretics (Venance et al., 2006; Statland et al., 2018). These treatments aim to avoid or reverse attack triggers and restore muscle function.

### 3.1 The main clinical treatments for HyperPP

*SCN4A* mutations account for more than 50% of HyperPP cases (Venance et al., 2006). HyperPP is a disorder that affects the sodium channels in muscle cells, leading to abnormal sodium influx and muscle depolarization. This results in symptoms of myotonia (Maggi et al., 2020) or PP (Hayward et al., 1999). Most HyperPP mutations occur in the DIII–DIV structural domain (inactivation gate) of the sodium channel (Rojas et al., 1991; Sillen et al., 1996). HyperPP causes temporary muscle weakness or paralysis when blood potassium levels are high. Usually, HyperPP does not require any medication, but some people may benefit from using an inhaler with salbutamol, which induces hyperpolarization of the muscle membrane through stimulation of Na^+^/K^+^ pumps, terminating acute episodes of paralysis (Hanna et al., 1998). To prevent HyperPP episodes, some drugs, including CAI drugs and thiazide diuretics (hydrochlorothiazide), can be used to keep blood potassium levels low. NormoPP is a subtype of HyperPP that occurs even when blood potassium levels are normal (Chinnery et al., 2002). In 2018, a study showed that hydrochlorothiazide was very effective in treating NormoPP caused by the T704M mutation of *SCN4A* without any side effects (Akaba et al., 2018). However, whether the therapeutic response of these drugs depends on the genetic cause of HyperPP is unclear.

### 3.2 Typical drug therapy for HypoPP

HypoPP2 is a subtype of HypoPP that accounts for 20% of cases and results from mutations in the *SCN4A* gene encoding the Na_V_1.4 channel (Jurkat-Rott et al., 2000). These mutations affect the S4 segment of the VSD, resulting in the left shift in the steady-state inactivation curve and enhancing voltage sensitivity (Groome et al., 2014), and cause longer episodes of weakness than those in HyperPP without NDM features (Matthews et al., 2009). The most striking physiological feature is flaccid skeletal muscle paralysis with reduced serum potassium levels. CAIs can prevent attacks, but acetazolamide (ACZ) might worsen PP in patients with diabetes and has little effect on HypoPP2 (Ikeda et al., 2002; Matthews et al., 2011). Potassium-sparing diuretics (eplerenone or spironolactone) are an alternative for patients who do not respond to or tolerate CAIs (Weber and Lehmann-Horn, 1993). However, the exact mechanism of the interaction of these drugs and Na_V_1.4 is unknown. Therefore, new genotype-based drugs are needed for better treatment (Tricarico et al., 2006).

## 4 Ranolazine may be the most promising drug for treating congenital myasthenic syndrome

Congenital myasthenic syndrome (CMS) is a group of rare disorders that affect the transmission of signals between nerve and muscle cells. One of the causes of CMS is mutations in ion channels. Habbout et al. (2016) reported a new case of CMS caused by a recessive mutation in the *SCN4A* gene, which leads to congenital myopathy with PP (Habbout et al., 2016). However, knowledge of how to effectively treat this type of CMS is limited. Larusso et al. (2019) explored the use of three drugs targeting sodium channels: Mex, lacosamide, and ranolazine. They tested these drugs on a mouse model of congenital hypotonia, a disorder that causes low muscle tone and weakness. Ranolazine was the most potent drug and had the fewest side effects, suggesting that it could be a promising candidate for treating CMS and other congenital myopathies (Lorusso et al., 2019).

## 5 Some toxins specifically target and block the VSD and PM of Nav1.4

Recent studies have revealed that various venomous compounds, such as scorpion α-toxins, can interact with specific Na_V_ channel subtypes in various biological systems (Bosmans and Tytgat, 2007). These toxins are commonly used to capture prey or for defense. Organisms employing these toxins include pufferfish, seaweed, scorpions, spiders, and conical snails (Maatuf et al., 2019). Some of these toxins are sodium ion channel blockers (De Bellis et al., 2017), and others are channel activators (Deuis et al., 2017). Among the most typical closed pore blockers are TTX and saxitoxin (STX) (West et al., 2002; Cervenka et al., 2010), as well as compounds that extend channel activation by altering gating after binding to the pore, such as veratridine (VTD). The VSD of DII was identified as a major component of the neurotoxin receptor site.

### 5.1 Neuropeptide toxins

TTX, a natural cyclic guanidinium salt neurotoxin, is a potent neurotoxin that blocks the sodium channels of nerve cells. The nine types of Na_V_ channels in mammals can be classified into two groups based on their sensitivity to TTX: TTX-sensitive (TTX-s) and TTX-resistant (TTX-r). The TTX-s channels (Na_V_1.1–Na_V_1.4, Na_V_1.6, and Na_V_1.7) are inhibited by low TTX concentrations, while the TTX-r channels (Na_V_1.5, Na_V_1.8, and Na_V_1.9) are unaffected by TTX or require very high doses to be blocked. In 1985, Gonoi et al. (1985) discovered that rat skeletal muscle cells had both types of channels, and they could be distinguished using colchicine to induce muscle ball formation. The skeletal muscle Na_V_1.4 channel was TTX-s, and its blockade by TTX prevented the generation of an action potential (Gonoi et al., 1985). TTX binding to the TTX-s channels depends on specific amino acid residues in the pore region of the channel, and mutations in these residues can reduce or abolish the affinity of TTX for the channel (Noda et al., 1989; Terlau et al., 1991; Boccaccio et al., 1999). In 1992, Satin et al. (1992) and Backx et al. (1992) showed that TTX binds with high affinity to channels that have aromatic residues (Phe or Tyr) at specific sites in the outer pore vestibule (Backx et al., 1992; Satin et al., 1992). Santarelli et al. (2007) investigated the role of these residues in the TTX-s Na_V_1.4 channel and found that they interacted directly with the guanidine group of TTX through π -cation bond attraction to conservative Tyr or Phe in the TTX-s Na_V_1.4 channel. This explains the preference of TTX for channels with aromatic residues and the mechanism of TTX sensitivity considering cation bonds.

STX is a biguanide neurotoxin that is synthesized by the marine dinoflagellate *Gymnodinium catenatum*. Similar to TTX, STX binds strongly to the external vestibule of the Na_V_ channel, blocking the permeation pathway and competing with TTX for the binding sites. However, they differ in that STX has post-repolarization blocking and frequency-dependent blocking effects and an increased blocking effect with higher frequency (Rando and Strichartz, 1986). Furthermore, STX exhibits shorter binding and dissociation rates (Lonnendonker, 1989). Notably, the latest STX derivatives identified by Pajouhesh et al. (2020) displayed promising analgesic activity in clinical settings. In addition, Duran-Riveroll et al. (2016) reported a benzoyl analog of STX and performed theoretical docking simulations of STX and the identified analog with two alternative three-dimensional models based on Na_V_1.4.

Spider venom is particularly enriched in Na_V_ modulators, but not all of them affect Na_V_1.4, e.g., hainantoxin-III (Zhang et al., 2021b). Generally, spider venom peptides interact with DIV to delay rapid inactivation, while peptides that bind with DI–III cause a voltage-dependent channel opening and closing shifts (Bosmans et al., 2008). Spider peptides are useful for identifying Na_V_1.4 and developing potential Na_V_ channelopathy therapies. A study by Moyer et al. (2018) showed that a 28-residue Ile mutant of the Na_V_1.7 toxin peptide JzTx-V (from the spider *Chilobrachys jingzhao*) had a 100-fold higher selectivity for the skeletal muscle Na_V_1.4 channel and blocked it effectively. Chen et al. (2020) observed that recombinant spider venom PaurTx-3 (rPaurTx-3) also inhibited Na_V_1.4 current with a half maximal inhibitory concentration (IC_50_) value of 61 nM. These spider venom peptides with Cys functional sites have unique pharmacological properties that make them attractive candidates for new therapies for skeletal muscle-nervous system diseases, as well as serving as drug precursors (Cardoso and Lewis, 2019).

Scorpion neurotoxins that target Na_V_ channels can be classified into two types: α- or β-toxins (Zhu et al., 2004). Under normal circumstances, α-toxins delay the fast inactivation of Na_V_ channels (Martin-Eauclaire et al., 2019). β-Scorpion toxins alter the activation threshold and reduce the peak current (Couraud et al., 1982). Researchers have been developing scorpion toxin derivatives for various purposes. For instance, Xu et al. (2020) modified the Trp38 residue of the purified scorpion toxin AGAP, a crucial residue for binding AGAP to the sodium channel, and found that the mutants reduced the inhibitory effects of AGAP on hNa_V_1.4 and analgesic effects on skeletal muscle. Additionally, Tz1, the main component of the venom from the Nerella scorpion (*Tityus zulianus*), alters the voltage dependence of Na_V_1.4 channel activation (Leipold et al., 2006), which could help identify other peptide toxins with pharmacological properties in scorpion venom.

μ-Conotoxin (μ-CTx), isolated from the venom of the cone snail (*Conus* spp.), has a unique structure with three disulfide bonds forming a type III cysteine motif (CC-C-C-CC). This toxin is an effective and selective blocker of the Na_V_ channel, wherein GIIIA, PIIIA, and SxIIIC selectively inhibit Na_V_1.4, mainly expressed in skeletal muscle, with IC_50_ values in the nanomolar range. Using μ-CTx GIIIA as a probe, Li et al. (2001) uncovered the clockwise orientation of the four structural domains of Na_V_ channels. Chen et al. (2014) investigated the effects of TTX, m-conotoxin DI-Asn181, and DIV-Glu172 on the Na_V_1.4 channel, which is involved in muscle contraction. They found that these toxins had different selectivity for the PIIIA isoform of Na_V_1.4, which is resistant to TTX. By introducing mutations (DI-N181R, DIV-E172Q) to the toxins, their affinity for PIIIA increased relative to that of TTX. A new μ-CTx toxin, SxIIIC, belonging to a class of conotoxins with hydroxyproline and high tissue specificity, was discovered by McMahon et al., in 2020. It can inhibit Na_V_1.4 in human skeletal muscle at an IC_50_ of approximately 15 nM but does not affect other Na_V_ channel subtypes and selectivity for Na_V_1.4 is about 10 times higher than that for other Na_V_ channels (MCMahon et al., 2020). Therefore, SxIIIC may be a promising drug candidate for diseases related to Na_V_1.4 dysfunction. The toxin-binding ion channel structure can help in the design of drugs targeting specific channels, and the toxin itself can be a useful drug or a model for drug development. We will discuss how toxins have inspired new ion channel toxins for pain relief. These modulators may also treat muscle and nerve disorders in the future.

### 5.2 Potent alkaloid toxins

Na_V_ channel biophysical properties are affected by different alkaloids. These alkaloids can alter specific Na channel functions and are useful as pharmacological probes to study the Na channel functional structure. Lipid-soluble toxins, such as grayanotoxin (GTX), batrachotoxin (BTX), VTD, and aconitine (AC), have some common characteristics: they bind to open Na channels, prevent Na channel inactivation, and shift the Na channel activation voltage to more hyperpolarized potentials (Khodorov, 1985; Narahashi and Herman, 1992). The binding sites of these toxins may include amino acids that are crucial for both activation and deactivation gating mechanisms.

BTX is a lipophilic steroidal alkaloid derived from the skin secretions of tree frogs (*Phyllobates* spp.). BTX affects Na_V_ channels by binding to their open state and shifting their activation voltage to more negative values, irreversibly promoting activation and inhibiting both rapid and slow inactivation. The binding site of BTX is in the inner pore region of Na_V_, where it prevents the necessary S6 rearrangement required for closing the channel after activation by binding at the level of its gated hinge residues, resulting in persistent sodium influx and muscle contraction (Li et al., 2002). Therefore, BTX is a potent toxin that inhibits the fast inactivation of rNa_V_1.4 (Bosmans et al., 2004; Logan et al., 2016). Ginsenoside Rg3 is a natural compound that can inhibit the effect of BTX on rNa_V_1.4 by competing for the same binding site. In 2008, Logan et al. (2016) showed that ginsenoside Rg3 reduced the BTX sensitivity of WT rNa_V_1.4 expressed in *Xenopus* oocytes, with an IC_50_ of 58.5 µM. They also identified a critical residue, L437, that was essential for both BTX and ginsenoside Rg3 binding. Mutating L437 abolished the ginsenoside Rg3 inhibition of rNa_V_1.4, indicating that L437 is a key determinant of the interaction between BTX and rNa_V_1.4 (Lee et al., 2008). Their study provides molecular insights into the mechanism of action of BTX and ginsenoside Rg3 on rNa_V_1.4 channels.

GTX is a type of diterpenoid compound that exists in the leaves, fine branches, and flowers of azaleas. Similar to BTX, GTX inhibits fast inactivation and alters ion selectivity, but it also reduces peak currents (Kimura et al., 2000). GTX has low potency with a median effect concentration (IC_50_) of 31 μM, and it affects the TTX-r Na^+^ channel (dorsal root ganglion cells) more than other sodium channels. However, GTX analogs have less impact on TTX-s and cardiac Na^+^ channels (Yakehiro et al., 1997; Yakehiro et al., 2000). Recent studies on GTX have focused on evaluating its activity against rNa_V_1.4 channels (Deuis et al., 2017).

VTD is a steroid-derived alkaloid from the roots of *Veratrum* of the Liliaceae family that affects sodium channels in cells. VTD can keep the Na_V_ channel open without repeated stimulation under voltage-clamp conditions (Ghatpande and Sikdar, 1999). Wang and Wang showed that VTD and LAs bind to similar sites inside the Na_V_ channel, and the receptor of bound VTD may be located in the internal vestibule, but the exact location is still unclear (Wang and Wang, 2003). Since the mutants were overly sensitive to the VTD inhibition of the Na_V_ peak current, they have been commonly used as Na_V_ channel activators for fluorescence identification (Vickery et al., 2004; Deuis et al., 2017).

AC is a steroid-derived alkaloid found in the plant *A. napellus*. It is a neurotoxin that binds to the neurotoxin-binding receptor site II on the Na_V_ α-subunit. This interaction prolongs the opening of the sodium channels, causing nerve stimulation and, eventually, paralysis (Rao and Sikdar, 2000). AC, renowned for its high cardiotoxicity, is often used to establish rat arrhythmia models (Zhao et al., 2013). A recent study by Imai et al. (2020) showed that goshajinkigan (GJG), an herbal medicine containing *A. napellus*, could inhibit Na_V_1.4 currents in C2C12 cells with an IC_50_ of approximately 73.13 µg/mL. They also reported that GJG could alleviate skeletal muscle stiffness and spasticity and speculated that Na_V_1.4 current inhibition was mainly due to AC (Imai et al., 2020). AC has similar effects on Na_V_1.4 and Na_V_1.5, but its activity on other Na_V_ subtypes needs further confirmation (Deuis et al., 2017).

## 6 Conclusion and outlook

In summary, Na_V_1.4 is the most prominent channel that regulates skeletal muscle contraction and is affected by various drugs and toxins (Table 2). The molecular structure of Na_V_1.4 has been partially elucidated, but more studies are required to reveal its biochemical, molecular, physiological, and pharmacological aspects. A better understanding of Na_V_1.4 function will facilitate the discovery of new therapies for Na_V_1.4 channelopathies that are specific and safe. Moreover, Na_V_1.4 modulators derived from drugs and toxins may have great potential as research tools or clinical agents. The results of this article contribute to recognition and awareness of ion channels and a deeper understanding of the importance of Na_V_ channels in mammalian bodies. Additionally, it will provide insights for the development of channel selective drugs and the prevention of ion channel diseases.

**TABLE 2:** Binding sites and efficacy of drugs and toxins.

	Drugs/Toxins	Type	Binding sites	IC_50_/EC_50_ (Na_V_1.4)
Anti-arrhythmics	Mex	Na_V_1.4 non-selective blocker	DIII S6—DIV S6 De Bellis et al. (2013)	IC_50_ = 256 ± 25 µM Farinato et al. (2019)
	Tocainide	Na_V_1.4 non-selective blocker	DIV S6 Imai et al. (2020)	IC_50_ = 580.7 ± 38 µM Talon et al. (2001)
	Flecainide	Na_V_1.4 non-selective blocker	DIII S6—DIV S6 De Bellis et al. (2013)	IC_50_ = 83.5 ± 17 µM Desaphy et al. (2004)
	Propafenone	Na_V_1.4 non-selective blocker	DIII S6—DIV S6 De Bellis et al. (2013)	IC_50_ = 18 ± 3 µM Farinato et al. (2019)
	Methocarbamol	NaV1.4 non-selective blocker	NA	IC_50_ ≈ 298 μM Suetterlin et al. (2015) (the muscle spindle)
	Ranolazine	Na_V_1.4 non-selective blocker	NA	NA
Local anesthetics	Lidocaine	Na_V_1.4 non-selective blocker	DIV S6 Ragsdale et al. (1994) DIII S4 and DIV S4 Sheets and Hanck (2007)	EC_50_ ≈ 20 µM Grant et al. (1989)
Antiepileptics	Lamotrigine	Na_V_1.4 non-selective blocker	DIV S6 Lipkind And Fozzard (2010)	NA
	Cannabidiol	Na_V_1.4 non-selective blocker	DI S6 and DII S6 Huang et al. (2021)	IC_50_ ≈ 10 μM Ghovanloo et al. (2021)
Neuropeptide toxins	Tetrodotoxin	Na_V_1.4 selective blocker	external to the SF Lee and Ruben (2008)	IC_50_ ≈ 10 nM Zimmer et al. (2014)
	Saxitoxin	Na_V_1.4 selective blocker	external to the SF Lipkind and Fozzard (1994)	IC_50_ ≈ 0.37 nM Moran et al. (2003)
	JzTx-V	Na_V_1.4 selective blocker	NA	IC_50_ ≈ 5.12 nM Luo et al. (2014)
	rPaurTx-3	Na_V_1.4 selective blocker	NA	IC_50_ ≈ 61 nM Chen et al. (2020)
	AGAP	Na_V_1.4 selective activator	PM and N-terminal domain Xu et al. (2020)	IC_50_ ≈ 10 nM Xu et al. (2020)
	Tz1	Na_V_1.4 selective activator	DII S3-S4 Cestele et al. (1998)	IC_50_ ≈ 8 μM Leipold et al. (2006)
	μ-CTx SxIIIC	Na_V_1.4 selective blocker	PM Pan et al. (2019)	IC_50_ ≈ 7 nM Walewska et al. (2008)
Potent alkaloids toxins	Batrachotoxin	Na_V_1.4 selective activator	PM Khodorov (1985)	IC_50_ ≈ 10 µM Logan et al. (2016)
	Ginsenoside Rg3	Na_V_1.4 selective blocker	DI S6 Lee et al. (2008)	IC_50_ ≈ 58.5 µM Wang and Wanf (2003)
	Grayanotoxin	Na_V_1.4 selective activator	DIV S6 Vickery et al. (2004)	IC_50_ ≈ 31 µM Rao and Sikdar (2000), Zhao et al. (2013)
	Veratridine	Na_V_1.4 selective blocker	DI S6, DII S6 and DIV S6 Wang and Wang (2003)	IC_50_ ≈ 55 µM Sheets et al. (2010)
	Aconitine	Na_V_1.4 selective activator	PM Zhu et al. (2020)	NA
	Goshajinkigan	NA	NA	IC_50_ ≈ 73 µg/mL Imai et al. (2020)

## References

[B1] Ahern C. A. Payandeh J. Bosmans F. Chanda B. (2016). The hitchhiker's guide to the voltage-gated sodium channel galaxy. J. Gen. Physiol. 147 (1), 1–24. 10.1085/jgp.201511492 26712848 PMC4692491

[B2] Akaba Y. Takahashi S. Sasaki Y. Kajino H. (2018). Successful treatment of normokalemic periodic paralysis with hydrochlorothiazide. Brain Dev. 40 (9), 833–836. 10.1016/j.braindev.2018.05.011 29907477

[B3] Alfonsi E. Merlo I. M. Tonini M. Ravaglia S. Brugnoni R. Gozzini A. (2007). Efficacy of propafenone in paramyotonia congenita. Neurology 68 (13), 1080–1081. 10.1212/01.wnl.0000257825.29703.e8 17389319

[B4] Andersen G. Hedermann G. Witting N. Duno M. Andersen H. Vissing J. (2017). The antimyotonic effect of lamotrigine in non-dystrophic myotonias: a double-blind randomized study. Brain 140 (9), 2295–2305. 10.1093/brain/awx192 29050397

[B5] Angsutararux P. Zhu W. Voelker T. L. Silva J. R. (2021). Molecular pathology of sodium channel beta-subunit variants. Front. Pharmacol. 12, 761275. 10.3389/fphar.2021.761275 34867379 PMC8640220

[B6] Backx P. H. Yue D. T. Lawrence J. H. Marban E. Tomaselli G. F. (1992). Molecular localization of an ion-binding site within the pore of mammalian sodium channels. Science 257 (5067), 248–251. 10.1126/science.1321496 1321496

[B7] Berghold V. M. Koko M. Berutti R. Plecko B. (2022). Case report: novel SCN4A variant associated with a severe congenital myasthenic syndrome/myopathy phenotype. Front. Pediatr. 10, 944784. 10.3389/fped.2022.944784 36090556 PMC9462513

[B8] Boccaccio A. Moran O. Imoto K. Conti F. (1999). Tonic and phasic tetrodotoxin block of sodium channels with point mutations in the outer pore region. Biophys. J. 77 (1), 229–240. 10.1016/S0006-3495(99)76884-0 10388752 PMC1300324

[B9] Bosmans F. Maertens C. Verdonck F. Tytgat J. (2004). The poison Dart frog's batrachotoxin modulates Nav1.8. FEBS Lett. 577 (1-2), 245–248. 10.1016/j.febslet.2004.10.017 15527793

[B10] Bosmans F. Martin-Eauclaire M. F. Swartz K. J. (2008). Deconstructing voltage sensor function and pharmacology in sodium channels. Nature 456 (7219), 202–208. 10.1038/nature07473 19005548 PMC2587061

[B11] Bosmans F. Tytgat J. (2007). Voltage-gated sodium channel modulation by scorpion alpha-toxins. Toxicon 49 (2), 142–158. 10.1016/j.toxicon.2006.09.023 17087986 PMC1808227

[B12] Buyan A. Whitfield A. A. Corry B. (2021). Differences in local anaesthetic and antiepileptic binding in the inactivated state of human sodium channel Nav1.4. Biophys. J. 120 (24), 5553–5563. 10.1016/j.bpj.2021.11.014 34774501 PMC8715241

[B13] Caietta E. Milh M. Sternberg D. Lépine A. Boulay C. McGonigal A. (2013). Diagnosis and outcome of SCN4A-related severe neonatal episodic laryngospasm (SNEL): 2 new cases. Pediatrics 132 (3), e784–e787. 10.1542/peds.2012-3065 23958773

[B14] Calhoun J. D. Isom L. L. (2014). The role of non-pore-forming β subunits in physiology and pathophysiology of voltage-gated sodium channels. Handb. Exp. Pharmacol. 221, 51–89. 10.1007/978-3-642-41588-3_4 24737232

[B15] Cannon S. C. (2018). Sodium channelopathies of skeletal muscle. Handb. Exp. Pharmacol. 246, 309–330. 10.1007/164_2017_52 28939973 PMC5866235

[B16] Cardoso F. C. Lewis R. J. (2019). Structure-function and therapeutic potential of spider venom-derived cysteine knot peptides targeting sodium channels. Front. Pharmacol. 10, 366. 10.3389/fphar.2019.00366 31031623 PMC6470632

[B17] Catterall W. A. (2000). From ionic currents to molecular mechanisms: the structure and function of voltage-gated sodium channels. Neuron 26 (1), 13–25. 10.1016/s0896-6273(00)81133-2 10798388

[B18] Catterall W. A. Goldin A. L. Waxman S. G. (2005). International Union of Pharmacology. XLVII. Nomenclature and structure-function relationships of voltage-gated sodium channels. Pharmacol. Rev. 57 (4), 397–409. 10.1124/pr.57.4.4 16382098

[B19] Catterall W. A. Lenaeus M. J. Gamal E. T. (2020). Structure and pharmacology of voltage-gated sodium and calcium channels. Annu. Rev. Pharmacol. Toxicol. 60, 133–154. 10.1146/annurev-pharmtox-010818-021757 31537174

[B20] Cervenka R. Zarrabi T. Lukacs P. Todt H. (2010). The outer vestibule of the Na+ channel-toxin receptor and modulator of permeation as well as gating. Mar. Drugs 8 (4), 1373–1393. 10.3390/md8041373 20479982 PMC2866490

[B21] Cestele S. Qu Y. Rogers J. C. Rochat H. Scheuer T. Catterall W. A. (1998). Voltage sensor-trapping: enhanced activation of sodium channels by beta-scorpion toxin bound to the S3-S4 loop in domain II. Neuron 21 (4), 919–931. 10.1016/s0896-6273(00)80606-6 9808476

[B22] Chanda B. Bezanilla F. (2002). Tracking voltage-dependent conformational changes in skeletal muscle sodium channel during activation. J. Gen. Physiol. 120 (5), 629–645. 10.1085/jgp.20028679 12407076 PMC2229551

[B23] Chen M. Peng S. Wang L. Yang L. Si Y. Zhou X. (2020). Recombinant PaurTx-3, a spider toxin, inhibits sodium channels and decreases membrane excitability in DRG neurons. Biochem. Biophys. Res. Commun. 533 (4), 958–964. 10.1016/j.bbrc.2020.09.103 33004176

[B24] Chen R. Robinson A. Chung S. H. (2014). Mechanism of mu-conotoxin PIIIA binding to the voltage-gated Na+ channel NaV1.4. PLoS One 9 (3), e93267. 10.1371/journal.pone.0093267 24676211 PMC3968119

[B25] Chinnery P. F. Walls T. J. Hanna M. G. Bates D. Fawcett P. R. W. (2002). Normokalemic periodic paralysis revisited: does it exist? Ann. Neurol. 52 (2), 251–252. 10.1002/ana.10257 12210802

[B26] Couraud F. Jover E. Dubois J. M. Rochat H. (1982). Two types of scorpion receptor sites, one related to the activation, the other to the inactivation of the action potential sodium channel. Toxicon 20 (1), 9–16. 10.1016/0041-0101(82)90138-6 6281941

[B27] De Bellis M. Boccanegra B. Cerchiara A. G. Imbrici P. De Luca A. (2023). Blockers of skeletal muscle Na(v)1.4 channels: from therapy of myotonic syndrome to molecular determinants of pharmacological action and back. Int. J. Mol. Sci. 24 (1), 857. 10.3390/ijms24010857 36614292 PMC9821513

[B28] De Bellis M. De Luca A. Desaphy J. F. Carbonara R. Heiny J. A. Kennedy A. (2013). Combined modifications of mexiletine pharmacophores for new lead blockers of Na(v)1.4 channels. Biophys. J. 104 (2), 344–354. 10.1016/j.bpj.2012.11.3830 23442856 PMC3552273

[B29] De Bellis M. Sanarica F. Carocci A. Lentini G. Pierno S. Rolland J. F. (2017). Dual action of mexiletine and its pyrroline derivatives as skeletal muscle sodium channel blockers and anti-oxidant compounds: toward novel therapeutic potential. Front. Pharmacol. 8, 907. 10.3389/fphar.2017.00907 29379434 PMC5770958

[B30] Decaen P. G. Yarov-Yarovoy V. Sharp E. M. Scheuer T. Catterall W. A. (2009). Sequential formation of ion pairs during activation of a sodium channel voltage sensor. Proc. Natl. Acad. Sci. U. S. A. 106 (52), 22498–22503. 10.1073/pnas.0912307106 20007787 PMC2799717

[B31] Decaen P. G. Yarov-Yarovoy V. Zhao Y. Scheuer T. Catterall W. A. (2008). Disulfide locking a sodium channel voltage sensor reveals ion pair formation during activation. Proc. Natl. Acad. Sci. U. S. A. 105 (39), 15142–15147. 10.1073/pnas.0806486105 18809926 PMC2567506

[B32] De Luca A. De Bellis M. Corbo F. Franchini C. Muraglia M. Catalano A. (2012). Searching for novel anti-myotonic agents: pharmacophore requirement for use-dependent block of skeletal muscle sodium channels by N-benzylated cyclic derivatives of tocainide. Neuromuscul. Disord. 22 (1), 56–65. 10.1016/j.nmd.2011.07.001 21802953 PMC3314985

[B33] De Luca A. Pierno S. Natuzzi F. Franchini C. Duranti A. Lentini G. (1997). Evaluation of the antimyotonic activity of mexiletine and some new analogs on sodium currents of single muscle fibers and on the abnormal excitability of the myotonic ADR mouse. J. Pharmacol. Exp. Ther. 282 (1), 93–100. 9223544

[B34] De Luca A. Talon S. De Bellis M. Desaphy J. F. Franchini C. Lentini G. (2003). Inhibition of skeletal muscle sodium currents by mexiletine analogues: specific hydrophobic interactions rather than lipophilia *per se* account for drug therapeutic profile. Naunyn Schmiedeb. Arch. Pharmacol. 367 (3), 318–327. 10.1007/s00210-002-0669-0 12644906

[B35] Desaphy J. F. Altamura C. Vicart S. Fontaine B. (2021). Targeted therapies for skeletal muscle ion channelopathies: systematic review and steps towards precision medicine. J. Neuromuscul. Dis. 8 (3), 357–381. 10.3233/JND-200582 33325393 PMC8203248

[B36] Desaphy J. F. De Luca A. Didonna M. P. George A. L. Camerino Conte D. (2004). Different flecainide sensitivity of hNav1.4 channels and myotonic mutants explained by state-dependent block. J. Physiol. 554 (Pt 2), 321–334. 10.1113/jphysiol.2003.046995 14608015 PMC1664778

[B37] Desaphy J. F. De Luca A. Tortorella P. De Vito D. George A. L. Conte Camerino D. (2001). Gating of myotonic Na channel mutants defines the response to mexiletine and a potent derivative. Neurology 57 (10), 1849–1857. 10.1212/wnl.57.10.1849 11723275

[B38] Deuis J. R. Mueller A. Israel M. R. Vetter I. (2017). The pharmacology of voltage-gated sodium channel activators. Neuropharmacology 127, 87–108. 10.1016/j.neuropharm.2017.04.014 28416444

[B39] Duran-Riveroll L. M. Cembella A. D. Band-Schmidt C. J. Bustillos-Guzmán J. J. Correa-Basurto J. (2016). Docking simulation of the binding interactions of saxitoxin analogs produced by the marine dinoflagellate Gymnodinium catenatum to the voltage-gated sodium channel NaV1.4. Toxins (Basel) 8 (5), 129. 10.3390/toxins8050129 27164145 PMC4885044

[B40] Eijkelkamp N. Linley J. E. Baker M. D. Minett M. S. Cregg R. Werdehausen R. (2012). Neurological perspectives on voltage-gated sodium channels. Brain 135 (Pt 9), 2585–2612. 10.1093/brain/aws225 22961543 PMC3437034

[B41] Fan C. Mao N. Lehmann-Horn F. Bürmann J. Jurkat-Rott K. (2017). Effects of S906T polymorphism on the severity of a novel borderline mutation I692M in Na(v) 1.4 cause periodic paralysis. Clin. Genet. 91 (6), 859–867. 10.1111/cge.12880 27714768

[B42] Farinato A. Altamura C. Imbrici P. Maggi L. Bernasconi P. Mantegazza R. (2019). Pharmacogenetics of myotonic hNav1.4 sodium channel variants situated near the fast inactivation gate. Pharmacol. Res. 141, 224–235. 10.1016/j.phrs.2019.01.004 30611854

[B43] Favre I. Moczydlowski E. Schild L. (1996). On the structural basis for ionic selectivity among Na+, K+, and Ca2+ in the voltage-gated sodium channel. Biophysical J. 71 (6), 3110–3125. 10.1016/S0006-3495(96)79505-X PMC1233800 8968582

[B44] Finsterer J. (2008). Primary periodic paralyses. Acta Neurol. Scand. 117 (3), 145–158. 10.1111/j.1600-0404.2007.00963.x 18031562

[B45] Gardill B. R. Rivera-Acevedo R. E. Tung C. C. Okon M. McIntosh L. P. Van Petegem F. (2018). The voltage-gated sodium channel EF-hands form an interaction with the III-IV linker that is disturbed by disease-causing mutations. Sci. Rep. 8 (1), 4483. 10.1038/s41598-018-22713-y 29540853 PMC5852250

[B46] Ghatpande A. S. Sikdar S. K. (1999). Voltage-dependent gating of veratridine-modified RIIA Na+ channel alpha subunit expressed heterologously in CHO cells. Pflugers Arch. 438 (3), 378–383. 10.1007/s004240050924 10398870

[B47] Ghovanloo M. R. Choudhury K. Bandaru T. S. Fouda M. A. Rayani K. Rusinova R. (2021). Cannabidiol inhibits the skeletal muscle Nav1.4 by blocking its pore and by altering membrane elasticity. J. Gen. Physiol. 153 (5), e202012701. 10.1085/jgp.202012701 33836525 PMC8042605

[B48] Gingrich K. J. Wagner L. N. (2016). Fast-onset lidocaine block of rat NaV1.4 channels suggests involvement of a second high-affinity open state. Biochim. Biophys. Acta 1858 (6), 1175–1188. 10.1016/j.bbamem.2016.02.033 26922882

[B49] Gonoi T. Sherman S. J. Catterall W. A. (1985). Voltage clamp analysis of tetrodotoxin-sensitive and -insensitive sodium channels in rat muscle cells developing *in vitro* . J. Neurosci. 5 (9), 2559–2564. 10.1523/JNEUROSCI.05-09-02559.1985 2411888 PMC6565308

[B50] Grant A. O. Dietz M. A. Gilliam F. R. Starmer C. F. (1989). Blockade of cardiac sodium channels by lidocaine. Single-channel analysis. Circ. Res. 65 (5), 1247–1262. 10.1161/01.res.65.5.1247 2553292

[B51] Groome J. R. Lehmann-Horn F. Fan C. Wolf M. Winston V. Merlini L. (2014). NaV1.4 mutations cause hypokalaemic periodic paralysis by disrupting IIIS4 movement during recovery. Brain 137 (Pt 4), 998–1008. 10.1093/brain/awu015 24549961 PMC3959555

[B52] Guy H. R. Seetharamulu P. (1986). Molecular model of the action potential sodium channel. Proc. Natl. Acad. Sci. U. S. A. 83 (2), 508–512. 10.1073/pnas.83.2.508 2417247 PMC322889

[B53] Habbout K. Poulin H. Rivier F. Giuliano S. Sternberg D. Fontaine B. (2016). A recessive Nav1.4 mutation underlies congenital myasthenic syndrome with periodic paralysis. Neurology 86 (2), 161–169. 10.1212/WNL.0000000000002264 26659129 PMC4731685

[B54] Hanna M. G. Stewart J. Schapira A. H. Wood N. W. Morgan-Hughes J. A. Murray N. M. (1998). Salbutamol treatment in a patient with hyperkalaemic periodic paralysis due to a mutation in the skeletal muscle sodium channel gene (SCN4A). J. Neurol. Neurosurg. Psychiatry 65 (2), 248–250. 10.1136/jnnp.65.2.248 9703181 PMC2170187

[B55] Hayward L. J. Sandoval G. M. Cannon S. C. (1999). Defective slow inactivation of sodium channels contributes to familial periodic paralysis. Neurology 52 (7), 1447–1453. 10.1212/wnl.52.7.1447 10227633

[B56] Huang C. W. Lai H. J. Lin P. C. Lee M. J. (2020). Changes of resurgent Na(+) currents in the Na(v)1.4 channel resulting from an SCN4A mutation contributing to sodium channel myotonia. Int. J. Mol. Sci. 21 (7), 2593. 10.3390/ijms21072593 32276507 PMC7177622

[B57] Huang C. W. Lin P. C. Chen J. L. Su W. C. Chang T. K. (2021). miRNA-148a enhances the treatment response of patients with rectal cancer to chemoradiation and promotes apoptosis by directly targeting *c-met* . Biomedicines 9 (9), 1371. 10.3390/biomedicines9101371 34680492 PMC8533359

[B58] Ikeda K. Iwasaki Y. Kinoshita M. Yabuki D. Igarashi O. Ichikawa Y. (2002). Acetazolamide-induced muscle weakness in hypokalemic periodic paralysis. Intern Med. 41 (9), 743–745. 10.2169/internalmedicine.41.743 12322805

[B59] Imai R. Horita S. Ono Y. Hagihara K. Shimizu M. Maejima Y. (2020). Goshajinkigan, a traditional Japanese medicine, suppresses voltage-gated sodium channel NaV1.4 currents in C2C12 cells. Biores Open Access 9 (1), 116–120. 10.1089/biores.2019.0034 32368413 PMC7194311

[B60] Jurkat-Rott K. Mitrovic N. Hang C. Kouzmekine A. Iaizzo P. Herzog J. (2000). Voltage-sensor sodium channel mutations cause hypokalemic periodic paralysis type 2 by enhanced inactivation and reduced current. Proc. Natl. Acad. Sci. U. S. A. 97 (17), 9549–9554. 10.1073/pnas.97.17.9549 10944223 PMC16902

[B61] Ke Q. Zhao Y. Li Y. Ye J. Tang S. (2022). Clinical comparison and functional study of the L703P: a recurrent mutation in human SCN4A that causes sodium channel myotonia. Neuromuscul. Disord. 32 (10), 811–819. 10.1016/j.nmd.2022.08.004 36050252

[B62] Khodorov B. I. (1985). Batrachotoxin as a tool to study voltage-sensitive sodium channels of excitable membranes. Prog. Biophys. Mol. Biol. 45 (2), 57–148. 10.1016/0079-6107(85)90005-7 2408296

[B63] Kimura T. Kinoshita E. Yamaoka K. Yuki T. Yakehiro M. Seyama I. (2000). On site of action of grayanotoxin in domain 4 segment 6 of rat skeletal muscle sodium channel. FEBS Lett. 465 (1), 18–22. 10.1016/s0014-5793(99)01715-9 10620699

[B64] Lee C. H. Ruben P. C. (2008). Interaction between voltage-gated sodium channels and the neurotoxin, tetrodotoxin. Channels (Austin) 2 (6), 407–412. 10.4161/chan.2.6.7429 19098433

[B65] Lee J. H. Lee B. H. Choi S. H. Yoon I. S. Shin T. J. Pyo M. K. (2008). Involvement of batrachotoxin binding sites in ginsenoside-mediated voltage-gated Na+ channel regulation. Brain Res. 1203, 61–67. 10.1016/j.brainres.2008.01.078 18321475

[B66] Lee M. J. Lin P. C. Lin M. H. Chiou H. Y. C. Wang K. Huang C. W. (2022). Kinetic alterations in resurgent sodium currents of mutant Na(v)1.4 channel in two patients affected by paramyotonia congenita. Biol. (Basel) 11 (4), 613. 10.3390/biology11040613 PMC9031228 35453812

[B67] Lee S. Y. Mackinnon R. (2004). A membrane-access mechanism of ion channel inhibition by voltage sensor toxins from spider venom. Nature 430 (6996), 232–235. 10.1038/nature02632 15241419

[B68] Leipold E. Hansel A. Borges A. Heinemann S. H. (2006). Subtype specificity of scorpion beta-toxin Tz1 interaction with voltage-gated sodium channels is determined by the pore loop of domain 3. Mol. Pharmacol. 70 (1), 340–347. 10.1124/mol.106.024034 16638971

[B69] Li H. L. Hadid D. Ragsdale D. S. (2002). The batrachotoxin receptor on the voltage-gated sodium channel is guarded by the channel activation gate. Mol. Pharmacol. 61 (4), 905–912. 10.1124/mol.61.4.905 11901230

[B70] Li R. A. Ennis I. L. French R. J. Dudley S. C. Tomaselli G. F. Marbán E. (2001). Clockwise domain arrangement of the sodium channel revealed by (mu)-conotoxin (GIIIA) docking orientation. J. Biol. Chem. 276 (14), 11072–11077. 10.1074/jbc.M010862200 11154701

[B71] Lipkind G. M. Fozzard H. A. (1994). A structural model of the tetrodotoxin and saxitoxin binding site of the Na+ channel. Biophys. J. 66 (1), 1–13. 10.1016/S0006-3495(94)80746-5 8130328 PMC1275657

[B72] Lipkind G. M. Fozzard H. A. (2005). Molecular modeling of local anesthetic drug binding by voltage-gated sodium channels. Mol. Pharmacol. 68 (6), 1611–1622. 10.1124/mol.105.014803 16174788

[B73] Lipkind G. M. Fozzard H. A. (2010). Molecular model of anticonvulsant drug binding to the voltage-gated sodium channel inner pore. Mol. Pharmacol. 78 (4), 631–638. 10.1124/mol.110.064683 20643904 PMC2981395

[B74] Logan M. M. Toma T. Thomas-Tran R. Du Bois J. (2016). Asymmetric synthesis of batrachotoxin: enantiomeric toxins show functional divergence against NaV. Science 354 (6314), 865–869. 10.1126/science.aag2981 27856903

[B75] Lonnendonker U. (1989). Use-dependent block of sodium channels in frog myelinated nerve by tetrodotoxin and saxitoxin at negative holding potentials. Biochim. Biophys. Acta 985 (2), 153–160. 10.1016/0005-2736(89)90360-x 2553115

[B76] Lorusso S. Kline D. Bartlett A. Freimer M. Agriesti J. Hawash A. A. (2019). Open-label trial of ranolazine for the treatment of paramyotonia congenita. Muscle Nerve 59 (2), 240–243. 10.1002/mus.26372 30390395 PMC6340713

[B77] Loussouarn G. Sternberg D. Nicole S. Marionneau C. Le Bouffant F. Toumaniantz G. (2015). Physiological and pathophysiological insights of Nav1.4 and Nav1.5 comparison. Front. Pharmacol. 6, 314. 10.3389/fphar.2015.00314 26834636 PMC4712308

[B78] Luo J. Zhang Y. Gong M. Lu S. Ma Y. Zeng X. (2014). Molecular surface of JZTX-V (β-Theraphotoxin-Cj2a) interacting with voltage-gated sodium channel subtype NaV1.4. Toxins (Basel) 6 (7), 2177–2193. 10.3390/toxins6072177 25055801 PMC4113750

[B79] Maatuf Y. Geron M. Priel A. (2019). The role of toxins in the pursuit for novel analgesics. Toxins (Basel) 11 (2), 131. 10.3390/toxins11020131 30813430 PMC6409898

[B80] Maggi L. Bonanno S. Altamura C. Desaphy J. F. (2021). Ion Channel gene mutations causing skeletal muscle disorders: pathomechanisms and opportunities for therapy. Cells 10 (6), 1521. 10.3390/cells10061521 34208776 PMC8234207

[B81] Maggi L. Brugnoni R. Canioni E. Tonin P. Saletti V. Sola P. (2020). Clinical and molecular spectrum of myotonia and periodic paralyses associated with mutations in SCN4A in a large cohort of Italian patients. Front. Neurol. 11, 646. 10.3389/fneur.2020.00646 32849172 PMC7403394

[B82] Marban E. Yamagishi T. Tomaselli G. F. (1998). Structure and function of voltage-gated sodium channels. J. Physiol. 508 (Pt 3), 647–657. 10.1111/j.1469-7793.1998.647bp.x 9518722 PMC2230911

[B83] Martin-Eauclaire M. F. Adi-Bessalem S. Hammoudi-Triki D. Laraba-Djebari F. Bougis P. E. (2019). Serotherapy against voltage-gated sodium channel-targeting αToxins from androctonus scorpion venom. Toxins (Basel) 11 (2), 63. 10.3390/toxins11020063 30678116 PMC6410273

[B84] Matthews E. Labrum R. Sweeney M. G. Sud R. Haworth A. Chinnery P. F. (2009). Voltage sensor charge loss accounts for most cases of hypokalemic periodic paralysis. Neurology 72 (18), 1544–1547. 10.1212/01.wnl.0000342387.65477.46 19118277 PMC2848101

[B85] Matthews E. Portaro S. Ke Q. Sud R. Haworth A. Davis M. B. (2011). Acetazolamide efficacy in hypokalemic periodic paralysis and the predictive role of genotype. Neurology 77 (22), 1960–1964. 10.1212/WNL.0b013e31823a0cb6 22094484 PMC3235354

[B86] Mcclatchey A. I. Van den Bergh P. Pericak-Vance M. A. Raskind W. Verellen C. McKenna-Yasek D. (1992). Temperature-sensitive mutations in the III-IV cytoplasmic loop region of the skeletal muscle sodium channel gene in paramyotonia congenita. Cell 68 (4), 769–774. 10.1016/0092-8674(92)90151-2 1310898

[B87] Mccusker E. C. Bagneris C. Naylor C. E. Cole A. R. D'Avanzo N. Nichols C. G. (2012). Structure of a bacterial voltage-gated sodium channel pore reveals mechanisms of opening and closing. Nat. Commun. 3, 1102. 10.1038/ncomms2077 23033078 PMC3493636

[B88] Mcmahon K. L. Tran H. Deuis J. R. Lewis R. J. Vetter I. Schroeder C. I. (2020). Discovery, pharmacological characterisation and NMR structure of the novel µ-conotoxin SxIIIC, a potent and irreversible NaV channel inhibitor. Biomedicines 8 (10), 391. 10.3390/biomedicines8100391 33023152 PMC7599555

[B89] Modoni A. D'Amico A. Primiano G. Capozzoli F. Desaphy J. F. Lo Monaco M. (2020). Long-term safety and usefulness of mexiletine in a large cohort of patients affected by non-dystrophic myotonias. Front. Neurol. 11, 300. 10.3389/fneur.2020.00300 32655465 PMC7326038

[B90] Moran O. Picollo A. Conti F. (2003). Tonic and phasic guanidinium toxin-block of skeletal muscle Na channels expressed in Mammalian cells. Biophys. J. 84 (5), 2999–3006. 10.1016/S0006-3495(03)70026-5 12719231 PMC1302862

[B91] Moyer B. D. Murray J. K. Ligutti J. Andrews K. Favreau P. Jordan J. B. (2018). Pharmacological characterization of potent and selective NaV1.7 inhibitors engineered from Chilobrachys jingzhao tarantula venom peptide JzTx-V. PLoS One 13 (5), e0196791. 10.1371/journal.pone.0196791 29723257 PMC5933747

[B92] Narahashi T. Herman M. D. (1992). Overview of toxins and drugs as tools to study excitable membrane ion channels: I. Voltage-activated channels. Methods Enzymol. 207, 620–643. 10.1016/0076-6879(92)07045-p 1326704

[B93] Nicole S. Fontaine B. (2015). Skeletal muscle sodium channelopathies. Curr. Opin. Neurol. 28 (5), 508–514. 10.1097/WCO.0000000000000238 26285000

[B94] Nitta J. Sunami A. Marumo F. Hiraoka M. (1992). States and sites of actions of flecainide on Guinea-pig cardiac sodium channels. Eur. J. Pharmacol. 214 (2-3), 191–197. 10.1016/0014-2999(92)90118-n 1325356

[B95] Noda M. Suzuki H. Numa S. Stühmer W. (1989). A single point mutation confers tetrodotoxin and saxitoxin insensitivity on the sodium channel II. FEBS Lett. 259 (1), 213–216. 10.1016/0014-5793(89)81531-5 2557243

[B96] OMalley H. A. Isom L. L. (2015). Sodium channel β subunits: emerging targets in channelopathies. Annu. Rev. Physiol. 77, 481–504. 10.1146/annurev-physiol-021014-071846 25668026 PMC4817109

[B97] Pajouhesh H. Beckley J. T. Delwig A. Hajare H. S. Luu G. Monteleone D. (2020). Discovery of a selective, state-independent inhibitor of Na(V)1.7 by modification of guanidinium toxins. Sci. Rep. 10 (1), 14791. 10.1038/s41598-020-71135-2 32908170 PMC7481244

[B98] Pan X. Li Z. Huang X. Huang G. Gao S. Shen H. (2019). Molecular basis for pore blockade of human Na(+) channel Na(v)1.2 by the mu-conotoxin KIIIA. Science 363 (6433), 1309–1313. 10.1126/science.aaw2999 30765605

[B99] Pan X. Li Z. Zhou Q. Shen H. Wu K. Huang X. (2018). Structure of the human voltage-gated sodium channel Nav1.4 in complex with β1. Science 362 (6412), eaau2486. 10.1126/science.aau2486 30190309

[B100] Patton D. E. West J. W. Catterall W. A. Goldin A. L. (1992). Amino acid residues required for fast Na(+)-channel inactivation: charge neutralizations and deletions in the III-IV linker. Proc. Natl. Acad. Sci. U. S. A. 89 (22), 10905–10909. 10.1073/pnas.89.22.10905 1332059 PMC50451

[B101] Payandeh J. Scheuer T. Zheng N. Catterall W. A. (2011). The crystal structure of a voltage-gated sodium channel. Nature 475 (7356), 353–358. 10.1038/nature10238 21743477 PMC3266868

[B102] Peschel A. Cardoso F. C. Walker A. A. Durek T. Stone M. R. L. Braga Emidio N. (2020). Two for the price of one: heterobivalent ligand design targeting two binding sites on voltage-gated sodium channels slows ligand dissociation and enhances potency. J. Med. Chem. 63 (21), 12773–12785. 10.1021/acs.jmedchem.0c01107 33078946 PMC7667638

[B103] Portaro S. Rodolico C. Sinicropi S. Musumeci O. Valenzise M. Toscano A. (2016). Flecainide-responsive myotonia permanens with SNEL onset: a new case and literature review. Pediatrics 137 (4), e20153289. 10.1542/peds.2015-3289 26944947

[B104] Qin F. Yan C. Patel R. Liu W. Dong E. (2006). Vitamins C and E attenuate apoptosis, beta-adrenergic receptor desensitization, and sarcoplasmic reticular Ca2+ ATPase downregulation after myocardial infarction. Free Radic. Biol. Med. 40 (10), 1827–1842. 10.1016/j.freeradbiomed.2006.01.019 16678021

[B105] Ragsdale D. S. Mcphee J. C. Scheuer T. Catterall W. A. (1994). Molecular determinants of state-dependent block of Na+ channels by local anesthetics. Science 265 (5179), 1724–1728. 10.1126/science.8085162 8085162

[B106] Ragsdale D. S. Mcphee J. C. Scheuer T. Catterall W. A. (1996). Common molecular determinants of local anesthetic, antiarrhythmic, and anticonvulsant block of voltage-gated Na+ channels. Proc. Natl. Acad. Sci. U. S. A. 93 (17), 9270–9275. 10.1073/pnas.93.17.9270 8799190 PMC38631

[B107] Rando T. A. Strichartz G. R. (1986). Saxitoxin blocks batrachotoxin-modified sodium channels in the node of Ranvier in a voltage-dependent manner. Biophys. J. 49 (3), 785–794. 10.1016/S0006-3495(86)83706-7 2421797 PMC1329526

[B108] Rao S. Sikdar S. K. (2000). Modification of alpha subunit of RIIA sodium channels by aconitine. Pflugers Arch. 439 (3), 349–355. 10.1007/s004249900121 10650987

[B109] Rojas C. V. Wang J. Z. Schwartz L. S. Hoffman E. P. Powell B. R. Brown R. H. (1991). A Met-to-Val mutation in the skeletal muscle Na+ channel alpha-subunit in hyperkalaemic periodic paralysis. Nature 354 (6352), 387–389. 10.1038/354387a0 1659668

[B110] Sanchez-Solano A. Islas A. A. Scior T. Paiz-Candia B. Millan-PerezPeña L. Salinas-Stefanon E. M. (2017). Characterization of specific allosteric effects of the Na+ channel β1 subunit on the Nav1.4 isoform. Eur. Biophys. J. 46 (5), 485–494. 10.1007/s00249-016-1193-3 28012039

[B111] Sansone V. A. Burge J. Mcdermott M. P. Smith P. C. Herr B. Tawil R. (2016). Randomized, placebo-controlled trials of dichlorphenamide in periodic paralysis. Neurology 86 (15), 1408–1416. 10.1212/WNL.0000000000002416 26865514 PMC4831040

[B112] Sansone V. Meola G. Links T. P. Panzeri M. Rose M. R. (2008). Treatment for periodic paralysis. Cochrane Database Syst. Rev. (1), CD5045. 10.1002/14651858.cd005045.pub2 18254068

[B113] Santarelli V. P. Eastwood A. L. Dougherty D. A. Horn R. Ahern C. A. (2007). A cation-pi interaction discriminates among sodium channels that are either sensitive or resistant to tetrodotoxin block. J. Biol. Chem. 282 (11), 8044–8051. 10.1074/jbc.M611334200 17237232

[B114] Satin J. Kyle J. W. Chen M. Bell P. Cribbs L. L. Fozzard H. A. (1992). A mutant of TTX-resistant cardiac sodium channels with TTX-sensitive properties. Science 256 (5060), 1202–1205. 10.1126/science.256.5060.1202 1375397

[B115] Scholz A. Kuboyama N. Hempelmann G. Vogel W. (1998). Complex blockade of TTX-resistant Na+ currents by lidocaine and bupivacaine reduce firing frequency in DRG neurons. J. Neurophysiol. 79 (4), 1746–1754. 10.1152/jn.1998.79.4.1746 9535944

[B116] Segawa K. Nishiyama M. Mori I. Kubota T. Takahashi M. P. (2023). Hyperkalemic periodic paralysis associated with a novel missense variant located in the inner pore of Nav1.4. Brain Dev. 45, 205–211. 10.1016/j.braindev.2022.12.003 36628799

[B117] Sharan K. B. Brian E. M. Robert M. O. Storer R. I. Swain N. A. (2015). Voltage gated sodium channels as drug discovery targets. Channels (Austin) 9, 360–366. 10.1080/19336950.2015.1079674 26646477 PMC4850042

[B118] Sheets M. F. Fozzard H. A. Lipkind G. M. Hanck D. A. (2010). Sodium channel molecular conformations and antiarrhythmic drug affinity. Trends Cardiovasc Med. 20 (1), 16–21. 10.1016/j.tcm.2010.03.002 20685573 PMC2917343

[B119] Sheets M. F. Hanck D. A. (2007). Outward stabilization of the S4 segments in domains III and IV enhances lidocaine block of sodium channels. J. Physiol. 582 (Pt 1), 317–334. 10.1113/jphysiol.2007.134262 17510181 PMC2075305

[B120] Sillen A. Wadelius C. Sundvall M. Ahlsten G. Gustavson K. H. (1996). Hyperkalemic periodic paralysis caused by recurring mutation in the adult muscle sodium channel alpha-subunit gene. Genet. Couns. 7 (4), 267–275. 8985730

[B121] Skov M. de Paoli F. V. Nielsen O. B. Pedersen T. H. (2017). The anti-convulsants lacosamide, lamotrigine, and rufinamide reduce myotonia in isolated human and rat skeletal muscle. Muscle Nerve 56 (1), 136–142. 10.1002/mus.25452 27783415

[B122] Soff G. A. Kadin M. E. (1987). Tocainide-induced reversible agranulocytosis and anemia. Arch. Intern Med. 147 (3), 598–599. 10.1001/archinte.147.3.598 3103562

[B123] Statland J. M. Bundy B. N. Wang Y. Rayan D. R. Trivedi J. R. Sansone V. A. (2012). Mexiletine for symptoms and signs of myotonia in nondystrophic myotonia: a randomized controlled trial. JAMA 308 (13), 1357–1365. 10.1001/jama.2012.12607 23032552 PMC3564227

[B124] Statland J. M. Fontaine B. Hanna M. G. Johnson N. E. Kissel J. T. Sansone V. A. (2018). Review of the diagnosis and treatment of periodic paralysis. Muscle Nerve 57 (4), 522–530. 10.1002/mus.26009 29125635 PMC5867231

[B125] Sternberg D. Maisonobe T. Jurkat-Rott K. Nicole S. Launay E. Chauveau D. (2001). Hypokalaemic periodic paralysis type 2 caused by mutations at codon 672 in the muscle sodium channel gene SCN4A. Brain 124 (Pt 6), 1091–1099. 10.1093/brain/124.6.1091 11353725

[B126] Streib E. W. (1987). Paramyotonia congenita: successful treatment with tocainide. Clinical and electrophysiologic findings in seven patients. Muscle Nerve 10 (2), 155–162. 10.1002/mus.880100209 3102961

[B127] Suetterlin K. J. Bugiardini E. Kaski J. P. Morrow J. M. Matthews E. Hanna M. G. (2015). Long-term safety and efficacy of mexiletine for patients with skeletal muscle channelopathies. JAMA Neurol. 72 (12), 1531–1533. 10.1001/jamaneurol.2015.2338 26658970

[B128] Talon S. De Luca A. De BELLIS M. Desaphy J. F. Lentini G. Scilimati A. (2001). Increased rigidity of the chiral centre of tocainide favours stereoselectivity and use-dependent block of skeletal muscle Na(+) channels enhancing the antimyotonic activity *in vivo* . Br. J. Pharmacol. 134 (7), 1523–1531. 10.1038/sj.bjp.0704366 11724759 PMC1573071

[B129] Terlau H. Heinemann S. H. Stuhmer W. Pusch M. Conti F. Imoto K. (1991). Mapping the site of block by tetrodotoxin and saxitoxin of sodium channel II. FEBS Lett. 293 (1-2), 93–96. 10.1016/0014-5793(91)81159-6 1660007

[B130] Tricarico D. Mele A. Conte C. D. (2006). Carbonic anhydrase inhibitors ameliorate the symptoms of hypokalaemic periodic paralysis in rats by opening the muscular Ca2+-activated-K+ channels. Neuromuscul. Disord. 16 (1), 39–45. 10.1016/j.nmd.2005.10.005 16368240

[B131] Ulbricht W. (2005). Sodium channel inactivation: molecular determinants and modulation. Physiol. Rev. 85 (4), 1271–1301. 10.1152/physrev.00024.2004 16183913

[B132] Venance S. L. Cannon S. C. Fialho D. Fontaine B. Hanna M. G. Ptacek L. J. (2006). The primary periodic paralyses: diagnosis, pathogenesis and treatment. Brain 129 (Pt 1), 8–17. 10.1093/brain/awh639 16195244

[B133] Vereb N. Montagnese F. Glaser D. Schoser B. (2021). Non-dystrophic myotonias: clinical and mutation spectrum of 70 German patients. J. Neurol. 268 (5), 1708–1720. 10.1007/s00415-020-10328-1 33263785 PMC8068660

[B134] Vickery R. G. Amagasu S. M. Chang R. Mai N. Kaufman E. Martin J. (2004). Comparison of the pharmacological properties of rat NaV1.8 with rat NaV1.2a and human NaV1.5 voltage-gated sodium channel subtypes using a membrane potential sensitive dye and FLIPRR. Recept Channels 10 (1), 11–23. 10.1080/10606820490270410 14769548

[B135] Walewska A. Skalicky J. J. Davis D. R. Zhang M. M. Lopez-Vera E. Watkins M. (2008). NMR-based mapping of disulfide bridges in cysteine-rich peptides: application to the mu-conotoxin SxIIIA. J. Am. Chem. Soc. 130 (43), 14280–14286. 10.1021/ja804303p 18831583 PMC2665793

[B136] Wang G. K. Wang S. Y. (2003). Veratridine block of rat skeletal muscle Nav1.4 sodium channels in the inner vestibule. J. Physiol. 548 (Pt 3), 667–675. 10.1113/jphysiol.2002.035469 12626674 PMC2342907

[B137] Watkins B. Schuster H. M. Gerwin L. Schoser B. Kröger S. (2022). The effect of methocarbamol and mexiletine on murine muscle spindle function. Muscle Nerve 66 (1), 96–105. 10.1002/mus.27546 35373353

[B138] Weber F. Lehmann-Horn F. (1993). Hypokalemic periodic paralysis. 20301512

[B139] West J. W. Patton D. E. Scheuer T. Wang Y. Goldin A. L. Catterall W. A. (1992). A cluster of hydrophobic amino acid residues required for fast Na(+)-channel inactivation. Proc. Natl. Acad. Sci. U. S. A. 89 (22), 10910–10914. 10.1073/pnas.89.22.10910 1332060 PMC50452

[B140] West P. J. Bulaj G. Garrett J. E. Olivera B. M. Yoshikami D. (2002). Mu-conotoxin SmIIIA, a potent inhibitor of tetrodotoxin-resistant sodium channels in amphibian sympathetic and sensory neurons. Biochemistry 41 (51), 15388–15393. 10.1021/bi0265628 12484778

[B141] Winters J. J. Isom L. L. (2016). Developmental and regulatory functions of Na(+) channel non-pore-forming β subunits. Curr. Top. Membr. 78, 315–351. 10.1016/bs.ctm.2016.07.003 27586289

[B142] Xu Y. Sun J. Yu Y. Kong X. Meng X. Liu Y. (2020). Trp: a conserved aromatic residue crucial to the interaction of a scorpion peptide with sodium channels. J. Biochem. 168 (6), 633–641. 10.1093/jb/mvaa088 32730584

[B143] Yakehiro M. Seyama I. Narahashi T. (1997). Kinetics of grayanotoxin evoked modification of sodium channels in squid giant axons. Pflugers Arch. 433 (4), 403–412. 9082327

[B144] Yakehiro M. Yuki T. Yamaoka K. Furue T. Mori Y. Imoto K. (2000). An analysis of the variations in potency of grayanotoxin analogs in modifying frog sodium channels of differing subtypes. Mol. Pharmacol. 58 (4), 692–700. 10.1124/mol.58.4.692 10999938

[B145] Zhang J. Mao W. Ren Y. Sun R. N. Yan N. Gong H. (2018). Simulating the ion permeation and ion selection for a eukaryotic voltage-gated sodium channel NaVPaS. Protein Cell 9 (6), 580–585. 10.1007/s13238-018-0522-y 29532417 PMC5966359

[B146] Zhang Y. Otto P. Qin L. Eiber N. Hashemolhosseini S. Kröger S. (2021a). Methocarbamol blocks muscular Na(v) 1.4 channels and decreases isometric force of mouse muscles. Muscle Nerve 63 (1), 141–150. 10.1002/mus.27087 33043468

[B147] Zhang Y. Wang L. Peng D. Zhang Q. Yang Q. Li J. (2021b). Engineering of highly potent and selective HNTX-III mutant against hNa(v)1.7 sodium channel for treatment of pain. J. Biol. Chem. 296, 100326. 10.1016/j.jbc.2021.100326 33493520 PMC7988488

[B148] Zhao Z. Yin Y. Wu H. Jiang M. Lou J. Bai G. (2013). Arctigenin, a potential anti-arrhythmic agent, inhibits aconitine-induced arrhythmia by regulating multi-ion channels. Cell Physiol. Biochem. 32 (5), 1342–1353. 10.1159/000354532 24280730

[B149] Zhu S. Bosmans F. Tytgat J. (2004). Adaptive evolution of scorpion sodium channel toxins. J. Mol. Evol. 58 (2), 145–153. 10.1007/s00239-003-2534-2 15042334

[B150] Zhu W. Li T. Silva J. R. Chen J. (2020). Conservation and divergence in NaChBac and Na(V)1.7 pharmacology reveals novel drug interaction mechanisms. Sci. Rep. 10 (1), 10730. 10.1038/s41598-020-67761-5 32612253 PMC7329812

[B151] Zimmer T. Haufe V. Blechschmidt S. (2014). Voltage-gated sodium channels in the mammalian heart. Glob. Cardiol. Sci. Pract. 2014 (4), 449–463. 10.5339/gcsp.2014.58 25780798 PMC4355518

